# The Role of Digital Media in Early Childhood Education and Care: A Qualitative Analysis of Educators’ Perceptions

**DOI:** 10.3390/bs16060970

**Published:** 2026-06-11

**Authors:** Josipa Jurić, Linda Podrug Krstulović, Ines Blažević

**Affiliations:** 1 Teacher Education, Faculty of Humanities and Social Sciences, University of Split, 21000 Split, Croatia; iblazevic@ffst.hr; 2 Kindergarten Marjan, 21000 Split, Croatia; lpkrstulovic@gmail.com

**Keywords:** digital media, early childhood education and care, educators’ perceptions, digital competence, children’s learning, qualitative research

## Abstract

Digital media is increasingly shaping the ways in which children learn, communicate, and participate in everyday activities from an early age. The aim of this study was to examine how educators in early childhood education and care perceive the role of digital media in children’s learning, behaviour, and development, with particular emphasis on patterns of use, educational potential, and the role of educators and parents in mediating children’s digital experiences. The study specifically contributes to understanding these issues within the Croatian preschool context, where qualitative research on educators’ everyday experiences with digital media remains limited. The study employed a qualitative approach using focus groups conducted with a sample of 20 female educators from Croatia, organised into four focus groups. The data were analysed using thematic analysis. The findings suggest that educators perceive digital media as a useful yet complex pedagogical tool whose value depends on the way it is used. A distinction was particularly evident between passive and active use of digital content, with active, guided, and purposeful use perceived as having greater educational potential. At the same time, educators also recognised the potential of digital media to support children’s learning, motivation, creativity, and engagement when integrated meaningfully into educational activities. Educators emphasized the importance of their own role in guiding children’s digital experiences, as well as the significant influence of the family environment on patterns of media use. They also highlighted challenges related to excessive screen exposure, the lack of clear pedagogical guidelines, and the need for additional professional support. The findings suggest the importance of strengthening educators’ digital competences, supporting collaboration with parents, and developing clearer pedagogical guidance for the use of digital media in early childhood education and care.

## 1. Introduction

### 1.1. Digital Media in Early Childhood Education

Digital media has become an integral part of children’s everyday lives from a very early age, significantly shaping their patterns of learning, play, and interaction with their environment (Alelaimat et al., 2020; Paulus et al., 2021). Nowadays, children grow up in environments abundant in digital content, where various devices, such as tablets, smartphones, and computers, are present in both family and institutional contexts and have become an integral part of their daily experiences (Huber et al., 2018; Teichert et al., 2021). Such early exposure to digital media creates a need for the development of digital literacy, which extends beyond technical skills and includes the ability to understand, interpret, and critically use digital content (He & Wen, 2025; Soyoof et al., 2024). In early childhood education and care, digital competence is developed through the purposeful and pedagogically guided use of information and communication technology across a range of activities. In the preschool context, digital tools support learning, documentation, and reflection, particularly when they encourage children’s active participation (Al-Abdullatif, 2022; Masoumi & Bourbour, 2023; Utanto & Pristiwati, 2024). Previous research indicates that educators most commonly use educational videos, digital picture books, music content, and interactive applications in their work with children. Such approach is consistent with the definition of digital competence as one of the key competences for lifelong learning, encompassing critical, responsible, and creative use of technology (European Parliament and Council of the European Union, 2006).

Numerous studies highlight the potential of digital media to support children’s development, particularly in the domains of language, cognition, and social skills, when integrated within developmentally appropriate and pedagogically designed contexts (Karimi & Lim, 2010; Plowman et al., 2012; Swargiary, 2024). The multimodal nature of digital content, which combines visual, auditory, and interactive elements, enables children to access information in diverse ways and can enhance their motivation and engagement in learning (Utami & Latiana, 2018). However, research emphasises that technology alone does not ensure high-quality learning. Its effectiveness depends on how it is used, the quality of the content, and the pedagogical context in which it is embedded (Riofrío-Calderón & Ramírez-Montoya, 2022).

### 1.2. Children’s Learning and Engagement in Digital Environments

In digital environments, children’s learning is often associated with increased levels of engagement, interactivity, and motivation, which can positively influence knowledge acquisition processes (Karimi & Lim, 2010; Swargiary, 2024). Interactive and game-like elements of digital content encourage children’s active participation and contribute to the development of attention, a sense of competence, and interest in learning (Arthur et al., 2001; Utami & Latiana, 2018). Children also integrate digital experiences into their everyday play, whereby digital media becomes part of the broader context of their development and social interaction (Teichert et al., 2021).

At the same time, research highlights the limitations and potential risks of digital environments. Children’s activities are often characterised by passive use of content, such as watching videos, which may reduce opportunities for active and in-depth learning (Huber et al., 2018). Furthermore, excessive or inappropriate use of digital media has been associated with difficulties in attention, behavioural regulation, and socio-emotional development (Paulus et al., 2021). For this reason, digital learning is increasingly viewed as a complex phenomenon that requires a balanced approach and an understanding of the interaction between the child, the content, and the context of use (Soyoof et al., 2024). In this regard, the distinction between passive consumption of content and active participation becomes crucial for understanding the educational potential of digital media.

### 1.3. The Role of Educators and Parents in Mediating Digital Learning

The role of adults, particularly parents and educators, is crucial in shaping children’s experiences with digital media and in determining their educational value (Qaiser, 2020; Smahelova et al., 2017). Through various mediation strategies, such as supervision, co-use, and guidance, parents influence the way children use digital technologies and the quality of their digital experiences (Soyoof et al., 2024; Teichert et al., 2021). Their attitudes and beliefs about digital media shape the home environment and can either support or constrain the development of children’s digital competences (Paulus et al., 2021).

In the institutional context, educators play a particularly important role, as they actively shape the everyday educational situations in which digital media is used. They not only determine the frequency and the purpose of technology use, but also the ways in which digital content is integrated into learning activities (Magen-Nagar & Firstater, 2019; Preradović et al., 2017). Their pedagogical decisions include assessing the appropriateness of content, directing children’s attention, encouraging interaction, and providing support when children encounter difficulties. Although research indicates that educators often hold positive attitudes towards digital technologies, their actual implementation in practice depends on the level of competence, the availability of resources, and opportunities for professional development (Utami & Latiana, 2018; Vidal-Hall et al., 2020). Particular emphasis is placed on the importance of pedagogical mediation, which enables digital media to become a tool for active, meaningful, and developmentally appropriate learning (Riofrío-Calderón & Ramírez-Montoya, 2022).

The Croatian preschool context is particularly relevant due to differences in institutional resources, digital infrastructure, and the still-developing pedagogical guidelines related to digital media use in early childhood education and care.

### 1.4. Aim of the Study

Despite the growing body of international research on digital media in early childhood education, findings continue to indicate the simultaneous presence of both potential benefits and risks associated with their use (Paulus et al., 2021; Teichert et al., 2021). Previous studies have increasingly explored educators’ perceptions and practices in different educational contexts; however, there is still a limited number of qualitative studies focusing on the Croatian early childhood education system and the specific pedagogical and institutional conditions within which digital media is integrated into everyday practice. This is particularly relevant given the contextual differences in the availability of digital resources, institutional support, pedagogical guidelines, and opportunities for professional development. In the Croatian context, research on digital media in early childhood education has predominantly focused on general attitudes or technological aspects, while less attention has been given to educators’ everyday experiences, reflections, and interpretations of digital media use in practice. Educators nevertheless play a key role in shaping children’s experiences with digital media within the institutional context.

The aim of this study is to examine how educators in early childhood education and care perceive the role of digital media in children’s learning, behaviour, and development, with particular emphasis on patterns of use, effects on children’s learning, and the role of educators and parents in mediating children’s digital experiences. The particular value of this approach is reflected in the fact that, unlike parents, educators observe children within a structured educational context and possess professional knowledge and experience that enable them to identify patterns of behaviour and learning within a broader developmental framework.

In accordance with the above, the following research questions were formulated:

- How do educators describe patterns of children’s digital media use, including the level of exposure, family context, and perceived effects on children’s behaviour and learning?
- How do educators assess the educational use and potential of digital media in preschool settings?
- How do educators perceive the role of educators and parents in guiding and mediating children’s digital experiences, including challenges, examples of good practice, and the need for additional support?

## 2. Methodology

### 2.1. Research Design

A qualitative descriptive research design was employed with the aim of gaining a deeper understanding of educators’ perceptions of the role of digital media in early childhood education and care. This approach proved to be suitable for exploring complex and contextually conditioned phenomena, such as educators’ everyday experiences and pedagogical practices. Particular emphasis was placed on the perspective of educators as professional and reflective observers of children’s behaviour and learning, who, through their professional experience and work within a structured educational environment, are able to identify patterns and differences in children’s use of digital media in a more systematic manner.

### 2.2. Participants

A total of 20 female educators employed in early childhood education and care institutions in Split-Dalmatia County in the Republic of Croatia participated in the study. Participation was voluntary, and the sample was formed using convenience sampling. The study was conducted in March 2026 through four focus groups, each comprising five educators. Participants differed in terms of years of professional experience, type of institution, educational group, and spatial context, enabling the inclusion of diverse perspectives on the use of digital media in work with children.

All participants were female, reflecting the gender structure commonly present in the Croatian early childhood education sector, where the profession is predominantly female. Although the sample was limited to one Croatian county and does not allow broader generalisation of the findings, the study aimed to provide an in-depth and contextually grounded understanding of educators’ experiences and perceptions rather than statistical representativeness.

The demographic and professional characteristics of participants are presented in Table 1.

The study was conducted in accordance with ethical principles for research in education. Prior to participation, the participants were informed about the aim and purpose of the study, the procedures for data collection and analysis, and their right to withdraw from the study at any time without consequences. Anonymity was ensured, and all data were used exclusively for research purposes. Audio recordings and transcripts were securely stored and were accessible only to the authors of the study.

### 2.3. Data Collection

Data were collected through audio recordings of four in-person focus groups (5 participants) conducted using a set of pre-prepared open-ended questions focused on the research topic. The focus groups included questions related to the presence of digital media in children’s lives, the ways in which they are used in the preschool context, their impact on children’s behaviour and learning, the role of educators and parents, as well as challenges and examples of good practice in the use of digital media. Each focus group lasted approximately 60 to 90 min and was moderated by the authors of the study, who have experience in educational research and qualitative data collection.

The focus groups were conducted in a quiet and familiar preschool setting in order to encourage open discussion and participant comfort. Throughout the data collection process, particular attention was paid to creating a supportive atmosphere in which all participants could express their views freely. Moderator bias was minimised through the use of pre-defined guiding questions, encouraging equal participation, and avoiding evaluative responses during discussions.

Data collection continued until thematic repetition and sufficient depth of responses were observed across focus groups, indicating data saturation.

### 2.4. Data Analysis

The collected data were analysed using reflexive thematic analysis following the approach proposed by Braun and Clarke (2006, 2021). Audio recordings of the focus groups were fully transcribed by the authors to ensure accuracy and familiarity with the data. Given the structure of the focus groups, the initial analytical framework was guided by the research questions; however, the analysis remained flexible and allowed for the emergence of new themes from the data. Therefore, the analysis combined both deductive and inductive elements.

The analysis was conducted in several phases: familiarisation with the data through repeated reading of the transcripts, initial coding of relevant text segments, organisation of codes into broader thematic categories, review and refinement of themes, and their final naming and interpretation. Coding and interpretation were conducted collaboratively by the authors through continuous discussion and comparison of interpretations in order to enhance analytical consistency and credibility. Rather than aiming for statistical intercoder agreement, the analysis followed a reflexive qualitative approach focused on interpretative depth and contextual understanding (Braun & Clarke, 2021).

Throughout the analytical process, analytical notes and reflections were documented to support transparency and reflexivity in the interpretation of the data. Particular attention was paid to preserving the authenticity of participants’ statements, and the interpretation of findings was grounded in recurring patterns and meanings emerging from the data.

## 3. Results

The results are presented in accordance with the three research questions. Each thematic unit contains several subsections that further elaborate specific aspects of educators’ perceptions and experiences related to children’s digital media use.

### 3.1. Analysis Related to Research Question 1

#### 3.1.1. Everyday Digital Media Use and Excessive Screen Exposure Among Children

The results indicate a very early and widespread presence of digital media in children’s lives. Educators emphasize that digital devices have become an integral part of everyday life, with children coming into contact with them from a very early age, most often in the family environment. One participant highlights that children “use them from birth, because parents use them”, thereby emphasizing the role of parents in shaping early digital habits.

At the same time, educators recognize the ambivalent nature of digital media, noting that they can have both positive and negative effects. As one participant states, “the presence of digital devices is divided—they can be as beneficial as they can be harmful”, with their constant availability to children identified as a particular concern. It is also emphasized that children today are “100% present” in digital environments and that there has been a significant increase in time spent in front of screens.

When referring to situations in which children come into contact with digital content, educators point out that this most often occurs within the family context, particularly during leisure time, before bedtime, or when parents need to attend to other responsibilities. One participant notes that children “most often come into contact during rest time for children and parents… simply to relax, the easiest thing is to switch on the TV or a phone/tablet for the child”. Digital media is therefore frequently used as a means of calming or entertaining children, further confirming their functional role in everyday family routines.

In addition to passive use, educators highlight that children increasingly use digital devices for play and learning, emphasizing their ability to quickly acquire digital skills: “children and parents use them, and this is how they get familiar with them through small games on tablets and mobile phones… they learn to handle them better than their parents”. Educators also observe significant changes in children’s digital habits in recent years, particularly increasingly early exposure to screens, more visually intense content, and shorter attention spans. As one participant notes, “children use devices earlier and earlier, content is faster and more visually intense, and attention is shorter”. A decrease in outdoor play and an increase in the use of digital devices indoors are also observed.

Educators further identify a range of behavioural and developmental indicators associated with excessive screen exposure. According to their observations, children who are more frequently exposed to digital media demonstrate increased focus on digital content, often “moving as close to the screen as possible” and showing a pronounced need for constant contact with devices. They are also described as watching content passively, “without following the content, but simply staring at the screen”.

Particular difficulties are observed in the areas of attention, emotional regulation, social interaction, and independent play. Educators report that such children “have shorter attention spans, less focus, difficulties with self-regulation, become frustrated more quickly, and find it harder to engage in longer activities”. They are often described as irritable and impulsive, especially when access to devices is restricted, and as more dependent on external stimulation: “they find it difficult to engage in play independently… because they need someone to entertain them”. Behavioural patterns such as restlessness, reduced creative play, lack of patience, and weaker group participation are also emphasized.

At the same time, educators note that children frequently transfer digital experiences into communication and play, as they “talk so much about what they have seen and process it in their minds… with their friends”. However, they also observe that children with lower exposure to digital media show greater engagement in activities, better communication, and more developed social skills, particularly in outdoor play and group interaction.

In situations involving excessive exposure or behavioural difficulties, educators report actively involving parents and, when necessary, specialist staff. They emphasize that excessive exposure is often linked to permissive parenting styles and a lack of clear boundaries, as parents frequently “give in because they know how the child will react if they do not get what they want”. According to educators, these patterns further complicate behaviour regulation in the preschool context and highlight the importance of consistent approaches within the family environment.

#### 3.1.2. Parents, Family Context, and Digital Content

The results indicate that educators consider the family context to be a key factor in shaping children’s digital habits. The role of parents is perceived as crucial, particularly in regulating screen time and selecting content. However, educators point out that parents often do not fully recognize the complexity of the issue, as they “do not particularly like this topic and tend to idealize the real situation”, which can make collaboration and the alignment of educational approaches more challenging.

Educators emphasize that parents have a significant influence on children’s digital habits, with differences between families arising from levels of education, life experience, and established boundaries. As they note, “parents are different, they devote different amounts of time to their children, and this is reflected in media use as well”, with particular emphasis on the observation that “older parents are more responsible and monitor it more closely”. It is also highlighted that parents play a key role in setting rules and guiding children. However, in practice, this role is often not consistently fulfilled.

Educators particularly emphasize the problem of a lack of control and clear boundaries in children’s use of digital media at home. According to their observations, children often have unrestricted access to devices and content: “parents give children media and inappropriate content without limits”, while early exposure to digital devices is also highlighted: “the child is getting dressed… he gives him the phone, when the child goes to the toilet… he gives him the phone”. Such examples point to the everyday integration of digital media into routine activities, but also to potentially inappropriate patterns of use. 

Differences between families in their approaches to digital media are also observed, with educators noting that “there are no rules” and that children come from very diverse backgrounds.

In terms of content, educators report that children most frequently use platforms such as “YouTube, TikTok, games”, as well as various digital games and applications. It is also noted that older children often influence younger ones, with more complex games being mentioned: “they have older brothers and sisters… they play Minecraft and Roblox”. This further confirms the influence of the social environment on content selection.

Educators estimate that children spend a significant amount of time in front of screens, often highlighting that “children mostly spend the whole afternoon in front of screens” and that this constitutes “a lot, a great deal” of time. It is also noted that some parents impose restrictions, while others allow unrestricted use: “some parents allow half an hour before bedtime, some allow unlimited use”. Such differences are further reflected in children’s behaviour in the preschool setting.

With the reference to the second research question, the following section presents the results related to the ways in which digital media is used in the preschool context and educators’ perceptions of their effects on children’s learning.

### 3.2. Analysis Related to Research Question 2

#### 3.2.1. Use of Digital Media in the Preschool Context

The results indicate that digital media in the preschool context is primarily used as a support for educational work rather than as a dominant form of activity. Educators emphasize that digital technologies are most often used to enrich content, present educational materials, and stimulate children’s interest in specific topics. As one participant states, “I use digital media in the preschool setting exclusively for educational purposes”, while another adds that they are used “mostly for educational purposes, but also for entertainment… but we use appropriate content”.

Digital media is used in work with children in various forms, including the presentation of images, videos, music, and educational applications. Educators point out that such content can facilitate the understanding of abstract or otherwise inaccessible phenomena (“I play a video song, a story… and then on the smart boards I have applications for learning letters, colours, sounds”). It is also emphasised that digital media enables the extension of children’s experiences, particularly in the context of exploration and documentation of activities.

Given the multimodal nature of digital content, the examples identified in educators’ responses were grouped according to the dominant modality through which children primarily experience the content, including visual, auditory, audio-visual, and interactive forms. Particular emphasis was placed on interactive content involving children’s active participation and engagement with digital materials. This categorization was used for descriptive and organizational purposes in presenting the findings, rather than as a formal multimodal analysis framework. The identified types of digital content used by educators in their work with children are presented in Table 2.

Technology is present in some urban preschools due to children with developmental difficulties: “we have smart boards because we also have children with difficulties, and everyone uses them…”. It is also verbalized that these children are much more dependent on screens: “some of our children need to undergo withdrawal from the use in order to be able to progress.” However, educators’ accounts reveal significant limitations in terms of the availability of technology and infrastructure. Some participants report that they do not have basic conditions for using digital media in their work: “there isn’t even internet in the preschool. I use my own internet and, if I want to explore something with the children… I use my phone.” It is also highlighted that educators often rely on their personal devices, indicating insufficient institutional support: “I have to have a laptop… we use it a lot (private preschool).” Based on educators’ accounts, the most common types of digital media used in work with children and in their everyday environment were identified and are presented in Table 3.

Despite these limitations, educators emphasize the importance of high-quality content and a selective approach to the use of digital media. One participant notes: “I think there is a lot of high-quality content that I could show to children, but I don’t have the means to do so”, further highlighting the gap between the potential of technology and the actual possibilities for its implementation.

With regard to decisions about the timing and manner of digital media use, educators stress that their use is planned in accordance with the aims of the activity and the needs of the children. Digital media is not used spontaneously, but rather as an additional tool to enrich content: “if an activity needs to be enriched with additional materials, then we include digital media as well”. The importance of balancing digital and other forms of activity is also emphasized: “digital media are not a substitute for play, but a support for learning”.

Educators also provide concrete examples of good practice, such as the use of digital tools for exploration, project-based learning, and the visualization of content: “it is planned… for example, as an introduction to an activity or a project, and then for deepening the topic”, as well as the use of digital platforms and applications for stimulating children’s interest and active participation.

In terms of rules and guidelines, the results indicate certain inconsistency in practice. Although there are international recommendations on the use of digital media, such as those issued by paediatric organizations, preschools do not have formal protocols in place, and educators rely on their own judgement: “there are no guidelines in the preschool; we rely on our own judgement”.

#### 3.2.2. Learning Through Digital Media

The results indicate that children use digital media for a variety of purposes, with activities such as watching videos, listening to music, and using educational applications being most prevalent. Educators note that children most often “watch short animated films… educational videos, listen to music”, but also engage in certain interactive activities when they are given access to such content. It is further emphasized that children acquire digital skills very quickly, as they “know how to ask for something to be played… or it comes automatically to them”, indicating a high level of competence in navigating digital environments. In order to provide a clearer insight into the ways digital media is used in preschool practice, Table 4 presents the types of digital tools, media, and activities reported by educators according to their primary educational purpose.

The use of digital media is strongly reflected in children’s behaviour, particularly in the areas of attention, emotions, and interaction. Educators note that during the use of digital devices, children demonstrate a high level of focus, as “their attention is focused, emotions vary, interaction—they withdraw from their surroundings”. Such attentional focus is often intense and, in some cases, almost complete, as reflected in the statement that “attention is 100% focused”. At the same time, it is observed that in these situations children may respond less to environmental stimuli and temporarily withdraw from social interaction.

In the context of learning, educators emphasize that digital media can have significant educational potential when used purposefully and with appropriate content. Through digital media, children acquire new knowledge and develop a range of competences, as “there can be educational games… and children form an understanding of the world, they learn about everything through media”. The importance of coordinating content with specific topics and activities is also highlighted: “the goal is the content… children really learn what the topic is about”. In addition to cognitive and language skills, educators recognize the development of digital competences, creativity, and social skills through digital activities.

A distinction between passive and active use of digital media is particularly emphasized. Passive viewing of content, according to educators’ perceptions, has a limited impact on learning, whereas active participation promotes greater engagement and understanding. As one participant notes, “it’s not just about looking at the screen, attention is constantly ‘refreshed’”, highlighting the importance of interaction with the content. It is also pointed out that children often transfer digital experiences into real-life contexts through play: “they watched how people build dominoes and then they built dominoes themselves”, indicating a process of linking digital and real-world experiences.

Educators further emphasize that learning is influenced not only by the amount of time spent in front of screens, but also by the type of content to which children are exposed. As they state, “it depends on the content… it depends on the type of content”, with high-quality, developmentally appropriate content having greater educational potential. However, it is also noted that excessive exposure and inappropriate content may have negative effects, particularly in relation to attention and behaviour, further emphasizing the need for careful content selection and the active role of adults in its interpretation. 

In line with the third research question, the following section presents the results related to educators’ perceptions of their role in mediating children’s digital experiences, as well as the challenges they encounter in practice, examples of good practice, and identified needs for additional support.

### 3.3. Analysis Related to Research Question 3: The Role of Educators, Challenges, and Support Needs in Children’s Digital Media Use

The results indicate that educators perceive their role in the context of digital media as complex and multidimensional, emphasizing their responsibility in guiding children’s use of technology. At the same time, some participants point out that their role is not dominant in comparison with the family context, and that they often find themselves dealing with the consequences of children’s digital habits: “we do not have a particularly strong role; rather, we are fighting a ‘battle’ with this problem of today”. Such statements suggest a perceived limitation of educators’ influence in relation to broader social and family circumstances.

Despite this, educators emphasize the importance of their role in setting boundaries, selecting content, and shaping children’s digital experiences. As they state, their task involves “guiding, setting boundaries, and selecting high-quality content, as well as developing a critical approach to media”, thereby highlighting the professional dimension of their role. Particular emphasis is placed on ensuring that children are not just passive users, but active participants in digital environments: “the goal is not to isolate children from digital media, but to use media purposefully and for children to be active users, rather than solely passive consumers”.

Educators also emphasize the importance of their own example and the thoughtful use of digital media in their work with children. As one participant notes, “I can use them in a controlled way and try to teach how to use digital media in a meaningful way”, highlighting the role of educators as behavioural models. In this context, digital media is not viewed as an end in themselves, but as a tool which, when used thoughtfully, can have educational value: “if something has interested the child, then it is meaningful, it becomes a source of learning”.

With regard to guiding children towards high-quality content, educators emphasize an active and planned role. Content is not left to chance, but is selected and offered to children in line with the aims of the activity: “we plan and provide it deliberately; we do not offer anything else”. The importance of encouraging an exploratory approach and the development of digital competences is also highlighted, including basic information-search skills: “I sometimes allow them, if we are working on a project… to explore those videos themselves”, but with the emphasis that children need to be taught “how to search the internet”.

At the same time, educators identify numerous challenges associated with digital media use in work with children, particularly difficulties related to attention, concentration, emotional regulation, and reduced engagement in activities without screens. As participants report, children show “frustration, reduced attention, a need for fast stimuli, and resistance to activities without screens”, while in activities requiring sustained focus they “definitely have difficulties with concentration and attention”. Educators also note that children often “do not show interest, concentration is very poor, attention is short”, and may appear disengaged during group activities. In response to these difficulties, educators emphasize the need to adapt activities through shorter and more dynamic content: “we start with shorter stories… then gradually extend the time… it is shorter for younger children”.

In addition to challenges related to children, participants highlight difficulties in cooperation with parents, particularly the lack of consistency and a shared approach. As they note, the issue is not only children’s behaviour, but also the broader context, whereby a “pedagogical approach includes communication, the structure of activities, and cooperation with parents”, indicating the need for a more systematic and coordinated approach.

Despite these challenges, educators also describe examples of good practice based on moderate, controlled, and purposeful use of digital media. Such approaches include “strictly moderate use and supervision of content, clear boundaries, controlled content”, as well as “limited and targeted use, high-quality content, joint viewing and discussion, encouraging play, movement, and social activities”. Educators further emphasize that digital media can have a positive impact when used in a planned manner and in accordance with children’s developmental needs, highlighting the importance of guidance, supervision, and teaching children to distinguish between beneficial and inappropriate content.

In their concluding reflections, educators emphasize the importance of limiting screen time in early childhood and ensuring supervision and active adult involvement: “limited time, supervision, and shared use”. They also stress the need for stronger institutional support, clearer guidelines, additional training, and better cooperation between educators, parents, and professional services. Although educators demonstrate a critical awareness of the challenges associated with digital media use, they nonetheless acknowledge its pedagogical potential and express a clear need for improved access to digital technologies and stronger institutional support to enable their meaningful and effective integration into practice.

## 4. Discussion

The findings of this study suggest that digital media in early childhood education and care cannot be viewed as inherently positive or negative, but rather as a complex pedagogical phenomenon whose effects depend on the way they are used, the context, and the involvement of adults. This understanding complies with research emphasising that digital media forms part of a broader educational environment that is continuously evolving under the influence of technological development (Nechakovska & Stojanovska, 2025; Plowman et al., 2012). The results of this study further suggest that educators recognise this complexity and interpret digital media in the context of everyday pedagogical practice, rather than as an isolated factor in learning. Similar perspectives have also been identified in Croatian studies emphasising the growing influence of media and digital technologies on children’s upbringing and development (Bilić, 2020; Blažević, 2012; Tolić, 2009).

In this context, a clear distinction between passive and active use of digital media is highlighted, which educators explicitly articulate. Active, interactive, and purposeful use is associated with greater educational potential, as supported by numerous studies highlighting the importance of engagement, exploration, and collaboration in digital environments (Al-Abdullatif, 2022; Karimi & Lim, 2010; Masoumi & Bourbour, 2023; Torres et al., 2021). In contrast, passive consumption of content, particularly in the absence of adult involvement, is associated with limited educational outcomes and weaker developmental effects, which is further supported by the findings of this study. Croatian authors have similarly warned about the risks of passive media consumption and the increasing normalisation of media use in children’s everyday routines (Blažević, 2012; Sindik & Veselinović, 2010).

At the same time, educators did not perceive digital media exclusively negatively, but also recognised their potential for supporting learning, motivation, creativity, and access to diverse educational content when used purposefully and under adult guidance.

The findings also point to a range of challenges associated with the use of digital media, particularly in the context of excessive screen exposure. Educators identify reduced attention, difficulties with self-regulation, and weaker social interaction among children who are more frequently exposed to digital content. These findings are consistent with research linking intensive use of digital media with behavioural and developmental difficulties (Kattein et al., 2023; Paulus et al., 2021; Torun Yeterge, 2025), as well as with studies from the field of health that highlight broader consequences, including sleep disturbances, reduced physical activity, and impacts on children’s overall development (Borges et al., 2025). Furthermore, paediatric research points to a lack of clear guidelines and the need for greater professional education regarding children’s digital exposure (Trevisani et al., 2025), which further emphasises the importance of a systematic approach to this area. Within the Croatian context, similar concerns regarding the influence of media on preschool children’s behaviour and development have been identified by Sindik and Veselinović (2010) and Sindik (2012), particularly in relation to the role of adults in regulating media exposure.

The findings of this study clearly suggest that children’s digital practices cannot be understood without insight into the family context. In accordance with previous research, parents play a key role in shaping children’s media habits through their own behavioural patterns, the regulation of time, and the selection of content (Rek & Kovačič, 2018; Tomora, 2025). It is also clear that parental stress and everyday responsibilities may contribute to the increased use of digital devices as a means of regulating children’s behaviour (Kattein et al., 2023), which is further supported by the observations of educators in this study. Croatian research likewise highlights that parents and educators often perceive media simultaneously as a useful educational tool and a source of developmental concern (Sindik, 2012).

Alongside the family context, educators also play a crucial role in shaping children’s digital experiences. The findings show that educators do not perceive digital media as a neutral tool, but rather as a pedagogical resource that requires thoughtful planning, guidance, and reflection. These findings are in accordance with research emphasising that educators’ beliefs, competences, and pedagogical decisions significantly influence the integration of digital media into the educational process (Aldhilan et al., 2025; Preradović et al., 2017; Utami & Latiana, 2018; Vidal-Esteve & Martín-Gómez, 2023; Vidal-Hall et al., 2020). This suggests that the mere presence of technology is not sufficient, but that its pedagogical interpretation and application are essential. This is particularly relevant in the Croatian preschool context, where institutional conditions, availability of digital infrastructure, and opportunities for professional development may differ between public and private institutions, as well as between urban and smaller communities.

It is particularly worth noting that educators in this study emphasise the need for a balance between digital and non-digital activities, viewing digital media as a complement rather than a substitute for play and direct interaction. This approach is consistent with research highlighting the importance of social and emotional learning and relationships in early childhood (Blewitt et al., 2021), as well as with theoretical perspectives emphasising experiential and social learning. In this sense, digital media has pedagogical value only when they are integrated into the broader context of the child’s development. Such findings are also aligned with Croatian discussions on media pedagogy, which emphasise the importance of critical, balanced, and developmentally appropriate media use in early childhood (Tolić, 2009; p. 97).

The findings can be further interpreted through the concept of digital well-being, which refers to a balance between the benefits and risks of digital use (Cao & Li, 2023). Although this concept is not yet clearly defined, the findings of this study contribute to its understanding through concrete pedagogical practices and educators’ experiences, thereby bridging the gap between theoretical approaches and real educational contexts.

Finally, the results highlight the importance of the broader institutional and social framework within which digital media is used. In accordance with previous research (Qaiser, 2020; Riofrío-Calderón & Ramírez-Montoya, 2022; Teichert et al., 2021; Utanto & Pristiwati, 2024), the effective integration of digital media requires a coordinated approach involving educators, parents, and educational policy, as well as continuous professional development. Educators in this study also emphasise the need for clearer guidelines and support, further supporting the importance of a systematic approach to digital education in early childhood. Similar studies also point to a lack of clear pedagogical frameworks and the need for ongoing professional support for educators in integrating digital technologies into everyday practice (Magen-Nagar & Firstater, 2019; Preradović et al., 2017; Utami & Latiana, 2018; Utanto & Pristiwati, 2024). In the Croatian context, these findings additionally point to the need for more systematic institutional support, clearer pedagogical guidelines, and stronger educator training systems related to digital competencies and media pedagogy (Peran & Raguž, 2019). Furthermore, Croatian methodological literature emphasises the value of focus groups in exploring complex educational and social phenomena through participants’ experiences and interpretations (Skoko & Benković, 2009), which further supports the qualitative approach used in this study.

## 5. Conclusions

### 5.1. Main Findings

The findings of this study suggest that digital media in early childhood education and care represents an important, yet complex pedagogical resource whose value largely depends on the way they are applied. A key role is attributed to educators, who, through their everyday pedagogical practices, content selection, and approaches to leading activities, actively shape children’s experiences with digital media. The study contributes by providing a contextualized insight into educators’ perceptions and everyday practices related to digital media use in preschool settings.

Furthermore, the findings point to the need to develop the digital competences of future educators, with particular emphasis on the pedagogically grounded and critical use of digital technologies in work with children. It is especially important to prepare them for working with new generations of children who are growing up in digitally saturated environments and who develop specific patterns of technology use from an early age. The findings additionally indicate the importance of integrating digital pedagogy and critical media use more systematically into educator training and professional development.

The pedagogical contribution of this study is reflected in emphasizing the importance of a thoughtful and balanced approach to digital media, where it is not viewed as a substitute for play, interaction, and experiential learning, but rather as a complement to them. The findings also point to the importance of distinguishing between passive and active use of digital content, as well as to the value of guided, purposeful, and developmentally appropriate use of digital tools in work with children.

### 5.2. Limitations of the Study and Implications for Future Research

This study has several limitations. First, the sample was limited to educators from one county in the Republic of Croatia, which restricts the transferability of the findings to broader contexts. Furthermore, data collection through focus groups proved demanding both organizationally and in terms of time, as it required coordinating the participation of a larger number of educators, which influenced the dynamics of the research process.

Future research could include larger and more diverse samples, as well as comparisons across different educational and cultural contexts. It would also be valuable to explore digital media use longitudinally and within primary education settings in order to better understand the continuity of children’s digital experiences across educational stages.

### 5.3. Practical Implications

Despite these limitations, the findings offer several implications for educational practice. The results highlight the importance of continuous professional development for educators in the area of digital competences and pedagogically grounded use of digital technologies. The findings also suggest the importance of strengthening cooperation between educators and parents in order to support more balanced and developmentally appropriate digital media use among children.

Rather than prescribing specific policy measures, the findings indicate the potential value of developing clearer pedagogical guidelines, parent support programmes, and institutional support systems related to digital media use in early childhood education and care. The findings additionally highlight the importance of providing educators and parents with clearer pedagogical guidance and opportunities for professional learning related to digital media use in early childhood education.

## Figures and Tables

**Table 1:** Demographic and Professional Characteristics of Participants.

Characteristic	Category	n
Gender	Female	20
Type of institution	Public preschool	15
	Private preschool	5
Educational group	Nursery groups	7
	Preschool groups	13
Spatial context	Urban areas	4 kindergartens
	Smaller communities	1 kindergarten
Years of professional experience	5–10 years	5
	11–15 years	4
	16–20 years	5
	21–25 years	3
	More than 25 years	3

**Table 2:** Digital Content Used in Preschool According to Modality.

Content Modality	Type of Content	Examples	Illustrative Quote
Visual content	Video content	Educational videos, animated films	children really learn what the topic is about
	Digital stories	Digital picture books, stories	I show a video, a song, a story
Auditory content	Musical content	Children’s songs, dance	we use music and songs in activities
Audio-visual content	Multimedia content	Educational videos with narration and sound	their attention is 100% focused
Interactive content	Digital platforms	eTwinning, YouTube and similar sources	we plan and provide it deliberately

**Table 3:** Types of Digital Media Used by Educators in Work with Children.

Type of Device	Examples	Illustrative Quote
Mobile devices	Smartphone, tablet	I use my own internet…on my phone
Computers	Laptop, desktop computer	I have to have a laptop… we use it a lot
Multimedia devices	Television, projector, interactive whiteboard	we have smart boards because we also have children with disabilities
Audio devices	CD player, Bluetooth speaker	we use songs and audio content with children

**Table 4:** Digital Tools, Media, and Activities Used in Preschool Practice According to Purpose of Use.

Purpose of Use	Type of Tool/Media	Examples	Illustrative Quote
Learning and content acquisition	Educational applications	Learning letters, colours, sounds	they have applications for learning letters, colours, sounds
	Digital materials	Educational videos, digital materials for exploring topics	children really learn what the topic is about
Play and engagement	Games	Simple educational games	they learn through small games on tablets and phones
	Interactive tools	Online quizzes, digital puzzles	their attention is constantly refreshed
Creative expression	Creative tools	Applications for creating animations and content	children should be active users
Presentation and communication	Presentation tools	Digital presentations, projects, display	we use them for introducing projects and topics
Documentation of activities	Digital media	Photographs and video recordings of activities	we document activities and children’s work

## Data Availability

The data presented in this study are available from the corresponding author upon reasonable request. The data are not publicly available due to privacy and ethical restrictions.
